# Algorithmic-Aided Approach for the Design and Evaluation of Curvilinear Steel Bar Structures of Unit Roofs

**DOI:** 10.3390/ma15103656

**Published:** 2022-05-20

**Authors:** Jolanta Dzwierzynska, Patrycja Lechwar

**Affiliations:** Faculty of Civil and Environmental Engineering and Architecture, Rzeszow University of Technology, Al. Powstancow Warszawy 12, 35-959 Rzeszow, Poland; p.lechwar@prz.edu.pl

**Keywords:** steel bar structures, parametric design, genetic algorithms, structural optimization, lightweight structures, manufacturing and processing

## Abstract

Rationalization in structural design in the field of steel structures mostly consists inreducing structural material. The aim of this work was to develop an algorithmic-aided, original and practical approach to shaping curvilinear steel bar structures of modular roofs, enabling their optimization. The first stage of shaping consists in creating algorithms that define the structures of shelters made of four roof units. Algorithmic definitions of the structures made it possible to obtain many variants of the roof structures with the adopted preliminary criteria. In order to evaluate the effectiveness of the individual variants, the genetic optimizations of the structures’ forms were carried out. Assuming that the structures were loaded with self-weights, the cross-sections of the structures’ members were optimized with the permissible deflections, while the structures’ weights were the optimization criteria. This allowed us to eliminate the design variants unfavorable in terms of shape and weight. In contrast, the structures with the most advantageous properties were then optimized for weight under snow and wind loads. The research allowed us to notice how the shapes of the structures influenced their efficiency. The dual approach proposed for shaping, which takes advantage of the generative design and consistent flow of information during shaping, allowed us to achieve better solutions compared to the traditional approach.

## 1. Introduction

At present, thanks to technological changes over the years, the production of steel is definitely better in terms of quality than the steel produced in the past. As a result of the development of material engineering, designers have at their disposal the steel of new grades not only with higher strength, but also steel that meets special requirements. The properties of steel and its advantages make it applicable in the design of various building structures, which encompasses bar, shell and tension structures [1]. However, the advantages of steel such as its high tensile and compressive strength as well as its ability to prefabricate are the reasons why it is widely used in the building industry [1]. Due to this fact, the rational shaping of steel structures deserves special attention.

The shaping of steel structures is defined as the progressive optimization of their shapes due to assumed criteria of strength. These criteria constitute a set of related rules concerning reliability [2], including bearing capacity, serviceability as well as resistance to exceptional environmental influences, which are mostly defined by standards [3,4,5,6]. Another important aspect of shaping steel bar structures is the calculation and design of joints [7]. Over the years, many different methodologies for shaping steel structures have been developed. Among them, the most popular are minimization of the energy used for producing structural material, minimization of material consumption, as well as material reuse [8]. 

However, the behavior of any structure depends not only on the kind of material used, acting loads and technological solutions, but also on the geometric form of it. Due to this fact, the so-called geometric shaping criteria of steel structures are also very important. The geometric shaping criteria mainly deal with geometric parameters of the module, the structure’s height and its span, as well as the characteristics of the structural members [9]. Some of these criteria have been determined in long-term practice as well as profound studies in the field of structural optimization. Nowadays, they are considered rational due to a present state of knowledge and the technology advancement level, as well as sustainable design [10]. 

In the old pre-computer age, the optimal shapes of the structures have been found by experiments such as, for example, inverted hanging models or soap film methods. The structures achieved by these experiments were of minimal surface shapes [11,12]. They were characterized by high stiffness with a minimal amount of bending, which was guaranteed by their forms. In general, shape optimization deals with the form or the contour of the structure; however, for the discrete bar structures it means the position of the nodes in the whole structure’s form.

Curvilinear steel bar structures are particularly demanding in terms of both shaping the geometric forms of them and establishing their topologies. However, the positions of bars in structures can be determined using both the numerical and geometric method. In the first method, in order to establish structure’s nodes, the coordinates of the grid points and the so-called directional vectors can be applied, whereas in the second method, the so-called base solids can be used, which divide a given space, or the so-called base surface, which is divided in order to obtain grids of bars [8].

Curvilinear steel bar structures require a more sophisticated approach to structural shaping as they can be realized with the minimal use of materials. In this case, the form and topology significantly influence efficiency of the structure and the flow of forces in it [13]. As it has been shown in previous publications, the geometry of the base surface, which is divided during shaping, has a significant impact on the properties of the structure formed on its basis [14]. Ruled surfaces, which are the surfaces composed of straight lines, are especially advantageous due to their discretization [15]. However, as far as ruled surfaces are concerned, the subgroup of them, the so-called Catalan surfaces, are the most popular for shaping roof structures [16]. However, considering the Catalan surfaces, the hyperbolic paraboloids have found the greatest application for shaping single or complex roof structures. This work deals with shaping curvilinear steel bar structures composed of the modules of Catalan surfaces as the units of a cylindroid or a conoid. It is a sequel to the authors’ previous study on the parametric design of roof structures composed of the repetitive modules of Catalan surfaces [16]. However, contrary to the previous publication, where reinforced concrete structures are concerned, here, steel bar structures are taken into consideration.

The way of shaping structures is also dependent on the shaping tools used, which change according to the development of technology. CAD and BIM software currently available on the market, apart from their basic purpose, i.e., digital recording of the structure, modeling and coding the entire information about it, are integrated with Computer Integrated Manufacturing [17,18]. However, modern tools for parametrically shaping structures such as ones working in Rhinoceros 3D environment, created by Robert McNeel and associates, present new possibilities for shaping [19]. Commonly, when new tools become available, the aspects of their proper usage arise. Noteworthy are the modern tools that allow algorithmic aided design, such as Revit (Dynamo) and Rhinoceros (Grasshopper). Thanks to this software, a structural model can be described using a script with variable parameters and then generated.

Algorithmic-aided structural shaping, which means the process when both the geometric model and structural analysis are realized by means of algorithms, is rather a new field of research. However, some studies explore the concept of algorithmic-aided structural shaping in architecture, engineering and construction [20,21,22,23,24]. The possibility of using genetic algorithms for the optimization of the structures being designed is explored in [25,26]. A genetic algorithm approach to designing non-linear steel frames with semi-rigid connections is presented in [27,28]. It deals with searching for a frame with the minimum weight by selecting the most suitable steel cross sections from a standard set. However, a generative design strategy for the minimum-weight design of diagrid tall buildings is proposed in [23].

Appropriate digital tools can facilitate the work of the constructor-designer and lead to the best solutions. In this context, the study aims at developing an original approach to shaping curvilinear steel bar structures of the modular roofs created from the repeating cylindroid and conoid surface units. This approach uses genetic algorithms as a tool to search for optimal solutions of the structures and for their evaluation. It also allows us to notice how the shapes of the structures influence their efficiency.

## 2. Shaping Approach

### 2.1. Possible Surface Discretization

The surface discretization method is very important. As mentioned previously, the load bearing capacity of each steel bar structure is directly related to the topology of the grid it constitutes. The discretization methods vary in the number of face edges, node complexity as well as face’s curvature. Another aspect regarding the discretization of the base surface for shaping a steel bar structure is the anticipated way of presenting this structure in the future, due to the fact that the steel bar structure can constitute an invisible supported system or be a visible steel-panel mesh. In the second case, the mesh directly determines the aesthetics of the shaped object, so its pattern should be of a high-quality. Triangulation of the surfaces is a common way to discretize surfaces. In general, triangular meshes are more rigid and stronger than the meshes of other types. However, quadrilateral or hexagonal meshes also have some structural advantages as they can be torsion free structures. On the other hand, in the case of the structures based on triangular meshes, six beams typically meet in one node. This means a significantly higher number of bar elements and node complexities compared to other types of meshes. Often, these disadvantages also result in low structural transparency, which can be an important feature of a see-through covering material such as glass or plastic polycarbonate sheets for roofing. Moreover, the per-area cost of triangular glass panels is higher than that of quadrilateral panels due to the fact that the weight of the structure is smaller when less steel is used. From this point of view, quadrilateral meshes are more optimal. On the other hand, triangulated grid structures can easily approximate free form shapes, which is not so easy in the case of the other meshes. The most commonly used method of curved surfaces discretization is so-called tessellation. This method involves the process of filling the surface or space with a repeated geometric motif that is usually a regular polygon. Various methods of surface tessellation are presented in [29].

Taking the above pros and cons into consideration, quadrilateral meshes will be applied on the surfaces constituting the roofs’ units. Additionally, a light polycarbonate material will be applied for roofing. Additionally, the planarity of the mashes’ cells will be tested in order to check the possibility of using planar panels.

### 2.2. The Shaping Tools Used

The most intensive development of various optimization methods in structure design began in the 1970s. It was related with the application of computer technology for solving various engineering problems regarding structural analysis, as well as material properties. Optimal shaping of steel bar structures has always been the necessary goal of structural engineering. Therefore, this problem has been the focus of scientists and practicing engineers and is reflected in numerous publications [24,30,31].

The optimization method applied often depends on the design tools used. In our research, Rhinoceros 3D software, was used, which is software equipped with visual scripting language. The software user needs to build an algorithm that is a graphical code, which is a fully functioning program. The idea of visual programming language is to replace the textual syntax with graphic objects in which are coded individual commands and data, as well as to create links between them. The Rhino scripts of the considered structures were created by means of Grasshopper and Karamba 3D [26].

Next, it has been applied so-called evolutionary optimization EO (genetic optimisation), which is based on a genetic algorithm inspired by the Darwinian law of natural selection. This process of optimization mimics the natural process for genetic coding and selection, as well as reproduction. The reason of it is to iteratively improve the individuals in each generation, according to a given optimization criteria associated with fitness functions. This is because EO deals with searching for optimal solutions in a population of possible variables, contrary to other optimization methods, which deal with improving one variable.

Nowadays, there is more and more interest in the application of EO in structural engineering. The genetic algorithm approach to structural topology optimization is applied in [32,33,34,35]. However, some attempts to design and optimize steel bar structures using visual scripts were undertaken in [24,36,37].

**Figure 1:**
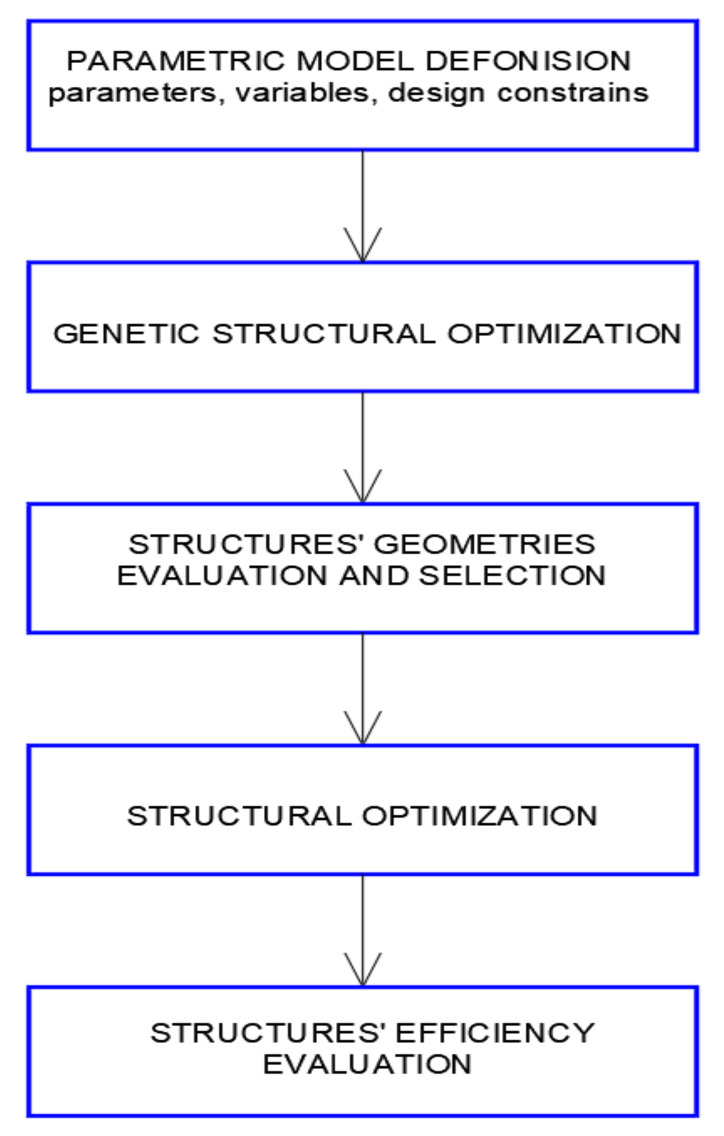
Basic scheme of the integrated shaping process.

In our case study, visual scripts were created in order to determine digital models of curvilinear steel bar structures as canopies composed of four modules of cylindroid shape or four modules of conoid shape. The structures have been defined by several geometric parameters. Next, the scripts describing structures’ geometries were extended in order to obtain structural models and connection properties, as well as loads applied. Then, the structures under self-loads were optimized due their masses with the application of EO. The results of the simulations were evaluated. After evaluation and the selection of the proper structures, their further optimization was carried out with the application of Robot Structural Analysis Professional software, assuming additional environmental loads acting on the structures [38]. A general structure shaping diagram is shown in Figure 1.

## 3. Results

As has been noted previously, due to their geometric properties, Catalan surfaces are applied as geometrical shapes for the formation of roofs [16,39]. However, the hyperbolic paraboloid, which is the surface of second order, is the most widely used. Due to their double curvature, hyperbolic paraboloids are exceptionally stiff and this is the reason for their common application. They are also widely discussed in the literature [24,37]. The cylindroid and the conoid are not as popular; however, they deserve attention as they can constitute interesting modular forms for the composition of compound roofs. Due to this fact, finite models of cylindroids and conoids have been considered as the roof’s units.

From a geometrical point of view, both the cylindroid and the conoid as the ruling surfaces can be defined as the surfaces composed of straight lines, the so-called surface rulings. Moreover, they can be determined by two generatrix lines and the so-called director plane to which all rulings are parallel. However, in the case of the cylindroid, both directrix lines are curved, whereas in the case of the conoid, one of the directrix lines is straight. Dependently on the shapes of the curved lines being surfaces’ directrix lines, various forms of the surfaces’ units and, thus, various forms of the composite roofs, can be obtained.

### 3.1. Geometric Modeling of Compound Unit Roofs by Grasshopper

**Figure 2:**
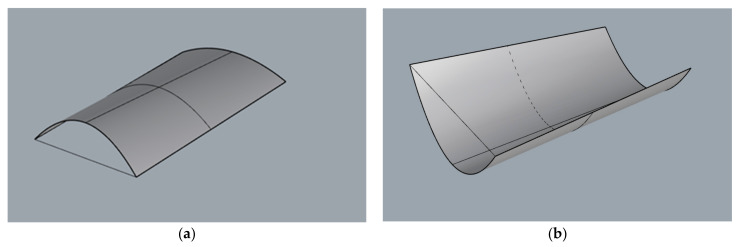
Example modules of cylindroid: (**a**) witch positive arch; (**b**) witch negative arch.

**Figure 3:**
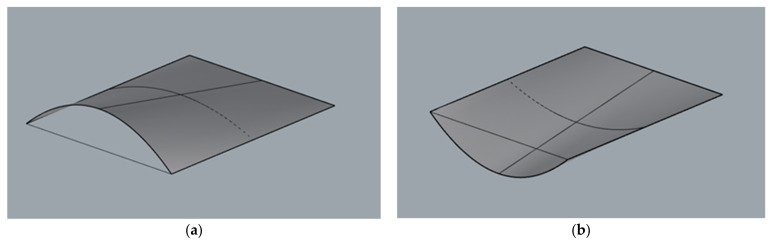
Example modules of conoid: (**a**) witch positive arch; (**b**) witch negative arch.

Each surface as a three-dimensional object can be described mathematically by a single equation with three space variables. In our case, in order to create a parametric surface by means of Grasshopper, each surface needs to be described by two parameters (u, v) measured along directrices and rulings. Parametric modelling began by establishing a series of points on two lines included in various planes and being the surfaces’ directrix lines. In the case of the cylindroid, both lines were curved and in the case of conoid, one of them was curved, whereas the second one was straight. The same number of points on each line were established, whereas the position of each point was described by parameter u. In this way, the surfaces’ rulings joined points of the lines, which corresponded to the same parameter value along u direction. As a result, the surface of the rulings was parallel to the same vertical director plane. The example modules of a cylindroid shape are presented in Figure 2, and the example modules of conoid shape are presented in Figure 3.

**Figure 4:**
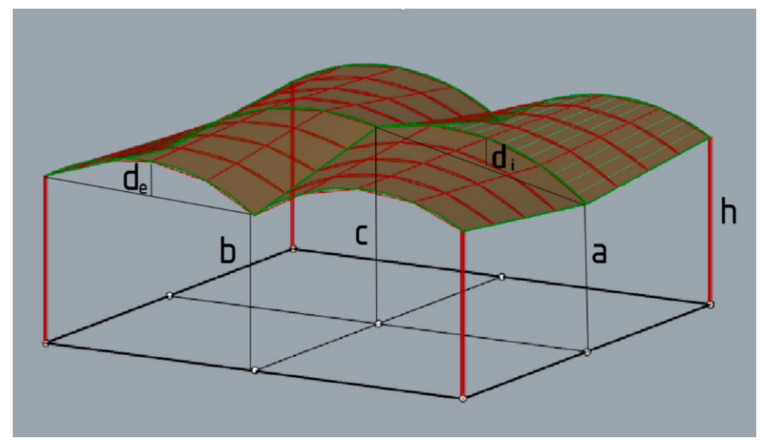
An example of the four-unit structure.

The shapes obtained constituted a base for creation of the four-module roofs supported by four columns. In the analyzed case, a sample square (9.6 m × 9.6 m) was covered with a roof consisting of four modules. However, due to the fact that the effective structures are sought, it has been assumed that each structure has two axes of symmetry, and it is composed of homogeneous modules of cylindroid grids or conoid grids. The geometry of the structure was described in an algorithmic way using Grasshopper’s visual script components. The example of a structure with markings of individual lengths is presented in Figure 4.

### 3.2. Geometric Modeling of Compound Unit Roofs by Grasshopper

Shaping of any steel bar structure can be treated as solving a problem according to the needed requirements and constrains. However, algorithmic-aided shaping is an action aimed at the logical description of a problem using a script and choosing the values of some structural parameters that meet the shaping criteria. The shaping criteria can be different; however, in the case of the steel bar structures, the main goal of shaping is structures’ masses reduction, unification of the structures’ elements, as well as using uncomplicated joints. They all influence a structural cost minimization.

The general scheme is shown in Figure 1 and applied to shape the considered curvilinear steel bar structures in order to reduce structural mass, encompassing two optimization processes. The first process was as follows: geometric modeling of the structures and establishing their structural models, which encompassed defining the shaping variables, identifying constrains, identifying the optimization criteria, optimizing the structures under dead loads and selecting the best solutions. The second process consisted of performing the structural analysis for the best roof forms, taking into account various load combinations, including environmental loads, optimization of structures due to their masses, selection and evaluation of the best results.

In order to perform the first optimization of the considered structures, the scripts determining the geometrical characteristics of the structures were developed by means of the Karamba 3D plug-in. For that reason, the bar grids’ vertices were transformed into structural nodes, the supports were defined as rigid and the structural joints were established: rigid for grid and hint joints connected the lattices with columns. However, in order to obtain a bar grid with a repeatable assortment of bars, an even division into four or five parts was assumed when discretizing the surface, which constituted one of the design variables. As a cover, it has been applied polycarbonate sheets attached to the bars with screws.

The simulations were performed with five different assumptions for both the roofs with cylindroid modules and the roofs with conoid modules, as listed below and according to the dimensional variables a, b, c, d_i_, d_e_ presented in Figure 4. However, the starting point for each simulation was the structure defined by the smallest parameters: a, b, c, d_i_, d_e_.

- **Case 1**—Cylindroid structure 1

Columns’ heights h = 4 m, the height of internal arches d_i_ = 0.7 m,
intervals of variables: a = 3–4 m, b = 3–4 m, c = 3–4 m, the height of the external arches d_e_ = −0.7–0.7 m,

- **Case 2**—Cylindroid structure 2

Columns’ heights h = 4 m, the height of the external arches d_e_ = 0.7 m, the height of internal arches d_i_ = −0.7 m,
intervals of variables: a = 3–4 m, b = 3–4 m, c = 3–4 m, the height of the internal arches d_i_ = −0.7–0.7 m

- **Case 3**—Cylindroid structure 3

Columns’ heights h = 3 m, the height of internal arches d_i_ = 0.7 m,
intervals of variables: a = 3–4 m, b = 3–4 m, c = 3–4 m, the height of the external arches d_e_ = −0.7–0.7 m,

- **Case 4**—Cylindroid structure 4

Columns’ heights h = 3 m, the height of the external arches d_e_ = 0.7 m,
intervals of variables: a = 3–4 m, b = 3–4 m, c = 3–4 m, the height of internal arches d_i_ = −0.7 m,

- **Case 5**—Cylindroid structure 5

Columns’ heights h = 3 m, the height of the external arches d_e_ = 0.7 m, the height of internal arches d_i_ = 0.7 m,
intervals of variables: a = 3–4 m, b = 3–4 m, c = 3–4 m,

- **Case 6**—Conoid structure 1

Columns’ heights h = 4 m, the height of the internal arches d_i_ = 0.7 m,
intervals of variables: a = 3–4 m, b = 3–4 m, c = 3–4 m,

- **Case 7**—Conoid structure 2

Columns’ heights h = 4 m, the height of the external arches d_e_ = −0.7 m,
intervals of variables: a = 3–4 m, b = 3–4 m, c = 3–4 m,

- **Case 8**—Conoid structure 3

Columns’ heights h = 3 m, the height of the internal arches d_i_ = 0.7 m,
intervals of variables: a = 3–4 m, b = 3–4 m, c = 3–4 m,

- **Case 9**—Conoid structure 4

Columns’ heights h = 3 m, the height of the internal arches d_e_ = −0.7 m,
intervals of variables: a = 3–4 m, b = 3–4 m, c = 3–4 m,

- **Case 10**—conoid structure 5

**Figure 5:**
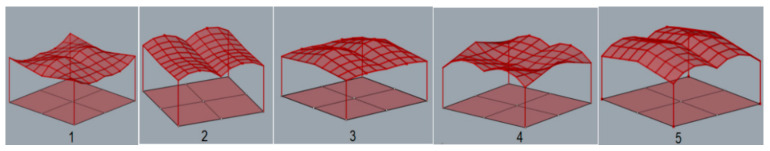
Various models of the roof structures composed of cylindroid units obtained due to simulations.

**Figure 6:**
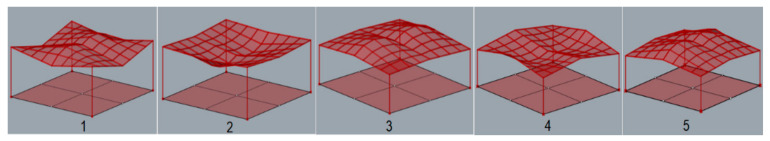
Various models of the roof structures composed of conoid units obtained due to simulations.

Columns’ heights h = 3 m, the height of the external arches d_i_ = 0.7 m,
intervals of variables: a = 3–4 m, b = 3–4 m, c = 3–4 m,

According to [1], maximum deflection for all structures was established equal to 3.8 cm. During the simulation, each structure was assumed to be loaded with its own weight and the structures’ masses were optimized taking into account the maximum allowable deflection. As the results of the performed simulations, five geometries of the roof structures composed of cylindroid units with the minimal masses, presented in Figure 5, and five roof structures with the conoid units with the minimal masses, presented in Figure 6, were established.

The dimensions which characterize the optimized roof structures with cylindroid units and with conoid units are given, respectively in Table 1 and Table 2.

**Figure 7:**
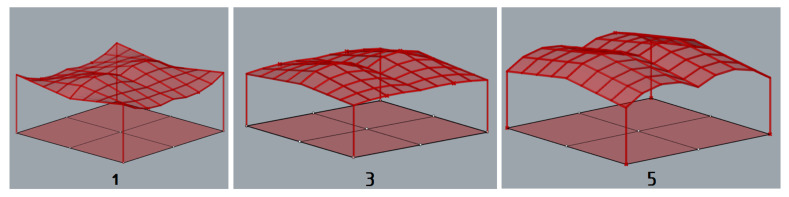
The models of the roof structures composed of cylindroid units favorable in terms of shape.

**Figure 8:**
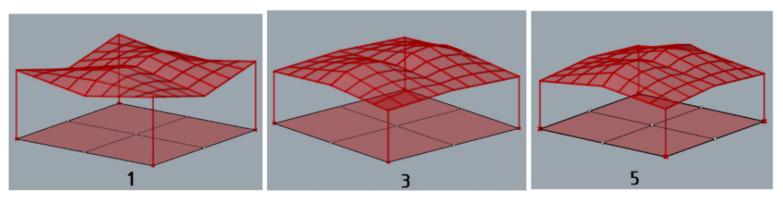
The models of the roof structures composed of conoid units favorable in terms of shape.

The structures obtained through simulations have been evaluated. The roofs that were unfavorable in terms of shape due to the possibility of snow or rainwater accumulation such as: the cylindroid structure 2, the cylindroid structure 4, the conoid structure 2, the conoid structure 4 were rejected. Thus, the following unit structures of favorable cylindroid shape were analyzed further: the cylindroid structure 1, the cylindroid structure 3, and the cylindroid structure 5, presented in Figure 7, as well as the following unit structures of favorable conoid shape: the conoid structure 1, the conoid structure 3, the conoid structure 5, are presented in Figure 8.

### 3.3. Structural Analysis by Means of Robot Structural Analysis Professional

Further analysis, optimization and dimensioning of the considered structures was carried out with the application of Robot structural Analysis Professional software. According to European Standards included in Eurocodes [3,4,5,6], both Ultimate Limit States (ULS) and Serviceability Limit States (SLS) have been verified during the process of shaping structures.

The selected structures were subjected to FEM analysis taking into account both permanent and environmental loads. Due to this fact, it has been assumed that each structure was loaded by permanent loads (self–weight), as well as environmental loads: snow load and wind load. The permanent load constituted cladding loads and structures’ loads. For the roof cladding, polycarbonate plastic sheets with a density of 0.02 kg/m^3^ and a panel thickness of 1.0 cm were used. The structural material steel of S 235 grade was used and the structural members with circular hollow cross-sections and wall thicknesses no less than 3.2 mm were also used. As far as environmental loads are concerned, the boundary conditions regarding snow and wind loads were assumed for the objects located in Rzeszow, Poland. Taking into account the third snow load zone location and the structures’ characteristics, it was assumed:

- characteristic value of snow load on the ground s_k_ = 1.2 kN/m^2^,
- the thermal coefficient C_t_ =1.0,
- the exposure coefficient C_e_ = 1.0.

Based on the standard guidelines and the symmetry of the structures, some simplifications have been proposed assuming the loads acting on the structures. However, in the performed analysis, two snow load cases have been considered: even snow load and the possibility of snowdrifts, whereas the roof’s shape coefficients have been assumed as for cylindrical roofs [5]. As far as the wind loads are concerned, it was assumed that base wind velocity pressure was equal to 0.3 kN/m^2^ and the loads were generated automatically, assuming several various wind directions [6].

The structures were optimized according to mass, taking into account the most unfavorable combination of loads. The analysis showed uneven stress distribution in individual elements of the structure. Therefore, in order to minimize the masses of the structures, their elements were divided into several groups for the purposes of dimensioning. The results of the structural analysis and the optimizations performed are presented in Table 3 and Table 4.

### 3.4. Analysis of the Possibility of Using Flat Panels on the Structure

**Figure 9:**
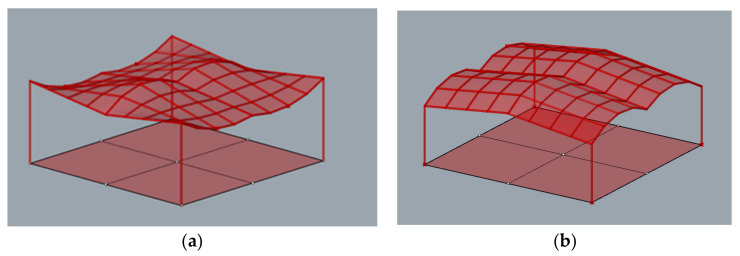
The structures with the possibility of using flat panels: (**a**) a cylindroid structure 1; (**b**) a cylindroid structure 5.

Another aspect which is very important in the case of the structures being analyzed is the possibility of using glass panels instead of the polycarbonate ones. The considered structures were analyzed with the use of lightweight polycarbonate panels, which minimized the total masses. Due to the easy adaptation of polycarbonate to any curved surface, the curvature of the panels used was not significant. When glass panels are used as planes, it is important for them to be flat quadrilaterals. Therefore, in order to check the planarity of individual panels of the structure, a verification algorithm was developed. The individual structures were verified in terms of the planarity of the polygons delineating the bar mesh. The most favorable shapes were found among the roofs of cylindroid shapes. Namely, in the case of the cylindroid structure 5, all flat panels can be applied, and in the case of the cylindroid structure 1, four flat panels can be applied in each roof unite, Figure 9.

It is worth mentioning that in the case of glass panels applied as roofing material, it is recommended to use stainless steel spider connections. However, depending on the node position, a one-, two- or four-point connector should be used.

## 4. Importance of Research

An algorithmic-aided method of efficiently shaping steel bar shelters with roofs composed of four units of cylindroid and conoid surfaces has been proposed.

**Figure 10:**
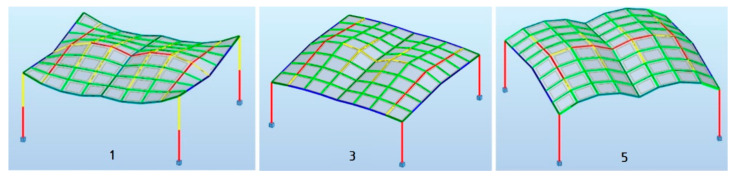
Maps on bars of the most efficient structures with cylindroid roofs.

**Figure 11:**
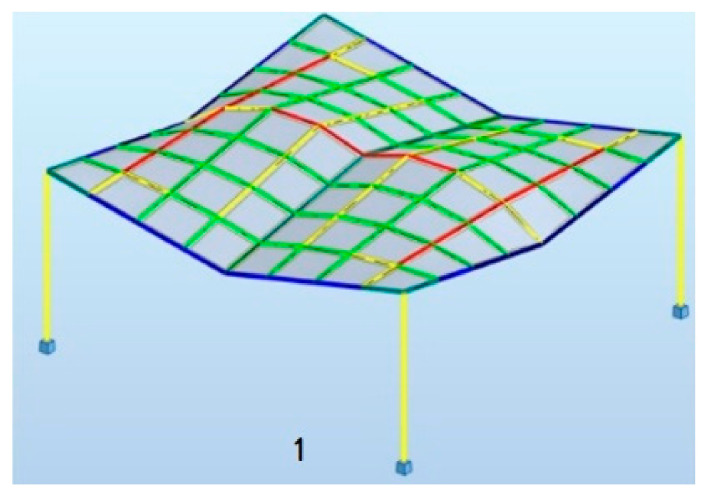
Maps on bars of the most efficient structure with conoid roof.

The developed visual scripts made it possible to test a large number of structures in the assumed specific dimensional ranges. The first stage of shaping, consisting of a algorithmically aided geometric design and structural design of the structures made it possible to distinguish several representative structures in terms of shape and mass. For this purpose, the optimization of each structure in terms of weight was carried out with a self-weight load, maintaining the permissible deflections. Based on the optimization results, five different geometries of the shelters with the roofs made of cylindroid units and five different geometries with the roofs made of conoid units were distinguished. The geometric characteristics of these structures are presented in Table 1 and Table 2. The shapes of the structures were evaluated and the structures with shapes unfavorable due to the possibility of snowfall accumulation were eliminated. The three most favorable structural geometries of the roofs with the cylindroid units and three with the conoid units were subjected to structural analysis assuming environmental loads and optimized due to their masses. The structures’ members were dimensioned. The summary of the results of these analyses are presented in Table 3 and Table 4 and show that, in general, the canopies with roofs made of cylindroid modules are lighter. On the other hand, among the structures with conoidal roofs, the structure called the conoid structure 1 is characterized by the smallest mass. The maps on bars for the most efficient structures with cylindroid modules: the cylindroid structure 1, structure 3, structure 5 are shown in Figure 10, whereas maps on bars on the most efficient structure 1 with conoid module are presented in Figure 11.

The above structures were designed to be applied as light material for roofing that is polycarbonate and easily adapts to curvilinear shapes. However, in the case of steel grid structures, it is convenient to use flat panels. In general, the grid structures created based on screw ruled surfaces as Catalan surfaces are characterized by no planar grid cells being spatial polygons. However, the grid roofs of the considered structures were tested in terms of using flat panels, e.g., glass. Thanks to the algorithm modification, the planarity of the grid cells were tested. The results of the analysis showed that among the structures tested, the polygons constituted the grid cells of the structures with cylindroid modules; that is, cylindroids 1 and cylindroid 5, are characterized by planarity. However, in the case of the structure called cylindroid 1, only four polygons of the bar grid of each module are flat. However, these modules are symmetrically located in the structure so the interesting solutions can be obtained. In contrast, in the case of the structure cylindroid 5, all of the lattice polygons are planar, so the entire roof can be covered with flat panels. Due to this fact, this structure seems to be the most efficient and universal.

The research showed that although the obtained structures due to optimization have two planes of symmetry, individual modules do not have such a property, therefore the obtained solutions are more effective than the initial assumptions of the simulation when symmetrical modules have been taken. The most efficient structural models both of cylindroid and conoid shape obtained as the result of the conducted research are economical, so they can be proposed as effective steel bar structures and they can find practical applications. Therefore, as the next step, laboratory tests are planned in order to verify the obtained results.

However, the research has shown that a holistic approach to the issue of shaping the steel bar structures, which is based on algorithmic aid, is appropriate, as it allows us to obtain optimal solutions within the assumed ranges of the design variables. Due to this fact, the research is worth continuing and extending in order to shape more complex structures, both single and modular ones.

In a broader sense, this research has shown how to apply generative tools to integrate structures’ geometries and structural efficiency as a single goal, in order to obtain optimal steel bar structures.

## 5. Conclusions

An algorithmic-aided method for shaping curvilinear steel bar structures of shelters with roofs composed of four grid shell units was proposed. The bar grids were created on the basis of the Catalan surface shapes, in particular the cylindroid and the conoid. The novelty of this method consists in:

- the use of algorithmically-aided structural analysis for the preliminary bars’ cross sections optimization and evaluation of the received solutions,
- a dual approach in shaping method which allows us to obtain optimal shapes of the steel bar structures in order to guarantee their effectiveness.

The applied method allowed us to:

- achieve the effective structures of both cylindroid and conoid shape in terms of load transfer and optimize them using mass as an optimization criterion,
- obtain the curvilinear steel bar structures with flat quadrilateral panels, which significantly influences the structural cost and permits the application of glass panels.

The authors plan to develop this method further. However, the achieved solutions and the developed method of shaping structures turned out to be promising, not only in terms of the possibility of structures’ optimization, but also as a design tool for searching for the efficient structures in the initial design phase.

## Figures and Tables

**Table 1:** Characteristic dimensions of various roof structures with cylindroid units.

Kind of the Structure	a [m]	b [m]	c [m]	d_e_ [m]	d_i_ [m]	h [m]
Cylindroid structure 1	3.28	3.39	3.89	−0.45	0.70	4.0
Cylindroid structure 2	3.7	3.86	3.66	0.70	0.36	4.0
Cylindroid structure 3	3.14	3.24	3.52	0.12	0.70	3.0
Cylindroid structure 4	3.47	4.00	3.08	0.70	−0.54	3.0
Cylindroid structure 5	3.0	3.60	3.30	0.70	0.70	3.0

**Table 2:** Characteristic dimensions of various roof structures with conoid units.

Kind of the Structure	a [m]	b [m]	c [m]	d_e_ [m]	d_i_ [m]	h [m]
Conoid structure 1	3.02	3.29	3.89	0.70	-	4.0
Conoid structure 2	3.74	3.81	3.10	−0.70	-	4.0
Conoid structure 3	3.61	3.63	3.85	-	0.70	3.0
Conoid structure 4	3.62	3.75	3.06	-	−0.70	3.0
Conoid structure 5	3.35	3.55	4.00	-	0.7	3.0

**Table 3:** The results of the structural analysis and optimization of the roof structures composed of cylindroid units.

Kind of the Structure	Max. Axial Force [kN]	Max. Bending Moment [kNm]	Max. Deformation [cm]	Structure’s Mass [kg]
Cylindroid structure 1	13.17	4.23	3.8	2308
Cylindroid structure 3	15.36	4.00	3.8	2223
Cylindroid structure 5	12.50	4.93	3.7	2272

**Table 4:** The results of the structural analysis and optimization of the roof structures composed of conoid units.

Kind of the Structure	Max. Axial Force [kN]	Max. Bending Moment [kNm]	Max. Deformation [cm]	Structure’s Mass [kg]
Conoid structure 1	14.55	5.07	3.4	2322
Conoid structure 3	26.32	5.00	3.7	2924
Conoid structure 5	12.76	7.98	3.7	3869

## Data Availability

Not applicable.
